# Femoroacetabular impingement–related deformities correlate with advanced osteoarthritis in asymptomatic caucasians over 60: A CT-based cross-sectional study

**DOI:** 10.1016/j.jor.2026.02.041

**Published:** 2026-02-10

**Authors:** Sophia Freya Ulrike Blum, Felix Schön, Katja Brauer, Petri Bellova, Jens-Peter Kühn, Ralf-Thorsten Hoffmann, Klaus-Peter Günther, Jens Goronzy

**Affiliations:** a Institute and Polyclinic for Diagnostic and Interventional Radiology, University Hospital Carl Gustav Carus, Technische Universität Dresden, Fetscherstraße 74, 01307, Dresden, Germany; b University Center of Orthopaedics and Traumatology, University Hospital Carl Gustav Carus, Technische Universität Dresden, Fetscherstraße 74, 01307, Dresden, Germany; c Center of Orthopaedics, Trauma Surgery and Rehabilitation Medicine, University of Greifswald, 17489, Greifswald, Germany

**Keywords:** Hip morphology, Femoroacetabular impingement, Osteoarthritis, Offset

## Abstract

**Objective:**

Osteoarthritis (OA) is a common finding in the elderly population. Femoroacetabular Impingement (FAI)-related deformities can cause OA. Their prevalence and clinical relevance in the asymptomatic elderly population are unclear. This study aims to determine the prevalence of cam and pincer deformities in asymptomatic, non-orthopedic Caucasian patients over 60 years in computed tomography (CT) and correlate the presence of those deformities with radiological signs of OA.

**Design:**

CTs of oncological patients over 60 years without any previous history of hip pathology or pain, containing a complete scan of both hips, were included. All hips were analysed for signs of OA according to the Kellgren-Lawrence (KL) classification. The acetabular anteversion, acetabular sector angles (ASA), and alpha angles were measured in clockwise positions of the hip. All angles were modeled conditionally on KL, sex, age, and obesity using random-effects median regression.

**Results:**

One hundred ninety-five non-orthopedic patients (130 male, 65 female; mean age 70.4 years; 290 hips) were included. Signs of severe OA (KL 3/4) were found in 19.2 %. Patients over 80 years had significantly higher alpha angles at 9-11 o'clock, higher ASA at 9-12 o'clock, and a higher acetabular anteversion at the level of the acetabular roof. Individuals with advanced OA showed higher alpha angles, higher ASA, and a lower acetabular anteversion than those without/with moderate OA.

**Conclusions:**

This study provides an estimate of the distribution of FAI-related deformities within a population of individuals over 60 years with asymptomatic hips. The prevalence of these deformities is correlated with advanced OA.

## 1 Introduction

In the general asymptomatic population, 6.5% show signs of osteoarthritis (OA) on radiographs.^1^ 7-12.6% of women over 65 show radiological signs of OA^2^, ^3^, ^4^, and 64.5% of them suffer from clinical symptoms such as pain.^3^ 7.4-37% of men over 65 show signs of OA.^2^^,^^5^ Accordingly, OA is a common secondary finding (0.9%-27%) on radiographs of the pelvis and hip obtained for non-orthopaedic disorders or in population-based epidemiological investigations.^2^ A long-term study based on radiographs reported an OA incidence of 11%^6^ in middle-aged women; a female preponderance has been reported in several studies^7^, ^8^, ^9^. Also, OA is defined as a multifactorial disease in which morphological deformities are among several major predisposing factors for cartilage degeneration and subsequent pathology. Developmental dysplasia of the hip (DDH), Perthes disease and slipped capital femoral epiphysis (SCFE) are established risk factors. More recently, subtle morphological abnormalities like cam-and pincer-type deformities causing femoroacetabular impingement (FAI) have been defined as prearthrotic deformities. Especially cam-type FAI can be associated with early cartilage degeneration and radiographic OA.^10^

A high rate of patients with femoroacetabular impingement are pain-free. A recent study on older men with signs of femoroacetabular impingement in radiographs showed an association with OA but not pain.^5^ The prevalence of FAI in young asymptomatic individuals has been investigated in multiple studies. Two-thirds revealed a Pincer-Deformity, and 37% showed a cam-type deformity.^11^ In patients with asymptomatic hips, radiological signs of FAI are found in 31–40%.^12^^,^^13^ All of these findings are based on radiographic evaluation. Computed tomography (CT) is superior to radiographs in identifying subtle signs of OA with circumscribed hip deformities.^14^

Especially in the scenario of secondary findings, it is essential to be aware of their prevalence and relevance in the clinical setting. We therefore sought to characterise the prevalence of typical femoroacetabular hip deformities in CT in a cohort of non-orthopaedic patients over 60 years with asymptomatic hips and analyse whether FAI-related deformities lead to more severe OA.

## 2 Material and methods

### 2.1 Patients and ethics

This study was approved by the local ethical board (EK 530122015, June 7, 2016). Caucasian patients over 60 years were consecutively asked if they wanted to participate in the study. They were all older than 60 years and were scheduled to undergo a complete CT scan of both hips for a non-orthopaedic indication, e.g., oncological staging. Written informed consent was obtained from all patients before the examination and interview. Exclusion criteria were as follows: patients without consent, patients with total hip arthroplasty (THA), and patients with acute or chronic pain. Abusive use of drugs and alcohol or inability to give informed consent to the study were also exclusion criteria.

### 2.2 Demographical data

One hundred ninety-five non-orthopaedic patients (130 male, 65 female; 390 hips) scheduled for a CT were included in this study. The mean age was 70.4 years (range, 60-88 years). Demographical data is shown in Table 1. Nearly half of the included patients were younger than 70 years (n = 92; 47%). The average BMI was 26.9 ± 5 kg/m^2^ with similar quantities in female and male volunteers. Only 23% (n = 47) of the patients had a BMI over 30 kg/m^2^.

**Table 1:** Demographics of the study population given in absolute number of patients and percent in parentheses.

	Overall, N = 195	Male, N = 130	Female, N = 65
**Age (years)**			
<69	92 (47%)	59 (45%)	33 (51%)
69-79	73 (37%)	54 (42%)	19 (29%)
>79	30 (15%)	17 (13%)	13 (20%)
**Adipositas (BMI>30kg/m2)**			
≤ 30	150 (77%)	104 (80%)	46 (71%)
> 30	45 (23%)	26 (20%)	19 (29%)
**American Society of Anesthesiologists physical status classification**			
0	0 (0%)	0 (0%)	0 (0%)
1	0 (0%)	0 (0%)	0 (0%)
2	6 (3.1%)	3 (2.3%)	3 (4.6%)
3	189 (97%)	127 (98%)	62 (95%)
4	0 (0%)	0 (0%)	0 (0%)
5	0 (0%)	0 (0%)	0 (0%)
**Reason for CT**			
Staging for oncological or chronic disease	155 (79%)	105 (81%)	50 (77%)
Staging for newly diagnosed tumour	2 (1.0%)	1 (0.8%)	1 (1.5%)
Preoperative imaging	4 (2.1%)	4 (3.1%)	0 (0%)
Further evaluation of suspicious findings	24 (12%)	13 (10%)	11 (17%)
Control scan under therapy	6 (3.1%)	5 (3.8%)	1 (1.5%)
Postoperative imaging	4 (2.1%)	2 (1.5%)	2 (3.1%)

### 2.3 Interview and clinical examination

To assess general risk factors for OA, the Body Mass Index (BMI, kg/m^2^) and the age of all patients were documented. The American Society of Anesthesiologists physical status classification and the reason for the performed CT were recorded to evaluate medical co-morbidities. Every patient underwent assessment with the Oxford Hip Score (OHS).

### 2.4 Assessment of OA and measures

All patients underwent CT with 1 mm slice thickness (Siemens Somatom Definition AS+, Erlangen, Germany). Images were post-processed as thick-slice maximum intensity projections (240 mm) to detect signs of osteoarthritis, analogous to the Kellgren and Lawrence (KL)^15^ classification system, as no conventional radiographs were available. Radiographic features used for OA grading included the presence of acetabular and femoral neck osteophytes, foveal osteophytes, acetabular and acetabular rim cysts, impingement cysts, and labral calcification, corresponding to established radiographic criteria of hip osteoarthritis. To date, no validated OA classification system exists for CT of the hip; therefore, use of the KL system facilitated comparability with prior studies based on conventional radiographs.

Additionally, all pelvises were analysed in bone window in a standardised 3D-reformation oriented along the centre of both femoral heads. The standardisation process has been published, with excellent inter- and intra-observer results.^16^^,^^17^ The acetabular anteversion was measured at the height of the joint centre and the roof of the acetabulum. The first slice showing the contour of the femoral head and opening of the acetabular contour when scrolling downwards was defined as the level of the acetabular roof. Modified acetabular sector angles (ASA) were measured in a clockwise manner from 9 o'clock (corresponding to the posterior section of the hip, regardless of the side) to 15 o'clock (9/10/11/12/13/14/15 o'clock). For all corresponding clockwise positions, the alpha angles between 9 and 15 o'clock were quantified. When acetabular osteophytes were present, the native acetabular contour was reconstructed using the “double-rim sign”, in which the original bone margin and the superimposed osteophytic margin produce a dual contour on imaging that serves as the anatomical reference 18, or targeting the bony base and cartilaginous cap arising from the periosteum at the junction between articular cartilage and bone, as defined by Turmezei et al.^19^ (Figure A.1). The femoral contour was identified likewise.^14^^,^^20^ Femoral torsion could not be measured because there was no additional scan over the femoral condyles.

### 2.5 Statistical methods

Patient and hip characteristics were described using absolute and relative frequencies for categorical variables and median and first/third quartiles for continuous variables. Distributions of alpha and ASA were visualised using violin plots.^21^ All angles were modeled conditionally on the KL score, sex, age, and obesity using random-effects median regression.^22^ Because of the highly skewed distributions of some angles, which could distort the results of ordinary linear regression or ANOVA, median regression was chosen. Since each patient was represented with two hips in our data, the median regression models included a random effect at the patient level to account for the correlation of angles within patients. In these regressions, standard errors were clustered at the patient level to account for within-patient correlation. Descriptive distributions are listed in Table 2, Table 3; model-based deviations are shown in Fig. 3. Statistical analysis was performed with R (version 4.1.2).

**Table 2:** Results of CT measurements (alpha angle, ASA, anteversion) in degrees expressed as median with 1st and 3rd quartiles in parentheses.

	Overall, N = 390	Male, N = 260	Female, N = 130	p-value
**Alpha angle**				
**15 o'clock**	42.0 (38.2, 47.6)	42.8 (39.4, 48.7)	40.4 (36.9, 44.8)	**<0.001**
**14 o'clock**	49.0 (44.0, 56.1)	50.8 (44.6, 57.8)	46.6 (42.3, 52.0)	**<0.001**
**13 o'clock**	57.6 (50.0, 67.7)	59.7 (52.2, 69.1)	52.7 (48.5, 61.6)	**<0.001**
**12 o'clock**	44.3 (38.3, 54.1)	46.2 (39.8, 60.2)	40.8 (36.7, 47.1)	**<0.001**
**11 o'clock**	43.0 (39.1, 74.6)	44.0 (40.2, 74.0)	41.3 (37.6, 79.7)	**0.027**
**10 o'clock**	41.0 (37.5, 53.3)	41.5 (38.0, 51.5)	40.0 (37.0, 72.0)	0.403
**9 o'clock**	43.8 (39.5, 54.9)	45.2 (39.6, 61.2)	42.2 (39.0, 47.1)	**0.033**
**ASA**				
**15 o'clock**	60.3 (54.7, 65.8)	60.9 (55.3, 67.2)	58.9 (53.0, 62.9)	**<0.001**
**14 o'clock**	82.8 (73.8, 94.1)	82.7 (74.1, 93.7)	83.2 (72.7, 94.8)	0.866
**13 o'clock**	120.0 (115.0, 124.9)	120.3 (115.1, 125.2)	119.8 (115.0, 124.1)	0.618
**12 o'clock**	129.5 (124.5, 133.6)	128.9 (123.7, 132.9)	130.4 (127.1, 134.5)	**0.002**
**11 o'clock**	127.7 (121.6, 133.1)	126.3 (120.5, 131.2)	130.7 (125.4, 136.0)	**<0.001**
**10 o'clock**	118.3 (112.8, 125.0)	116.5 (110.8, 122.1)	123.1 (116.7, 128.4)	**<0.001**
**9 o'clock**	102.2 (96.5, 108.9)	99.9 (94.6, 106.4)	107.3 (101.6, 113.0)	**<0.001**
**Anteversion**				
**femoral head centre**	21.0 (17.7, 24.4)	19.9 (16.6, 22.3)	24.2 (20.2, 28.7)	**<0.001**
**acetabular roof**	12.0 (6.8, 18.2)	10.2 (5.2, 16.0)	16.0 (11.1, 21.8)	**<0.001**

**Table 3:** Distribution of alpha angle, ASA, and anteversion assigned to Kellgren Lawrence Scores (KL). Angles are expressed as median with 1st and 3rd quartiles in parentheses.

	KL 0-1, N = 191	KL 2, N = 124	KL 3-4, N = 75	p-value
**Alpha angle**				
**15 o'clock**	40.9 (37.5, 46.3)	42.0 (38.4, 45.5)	44.5 (41.1, 53.2)	**<0.001**
**14 o'clock**	48.5 (43.6, 54.4)	48.6 (42.9, 55.5)	51.5 (46.0, 65.5)	**0.010**
**13 o'clock**	55.4 (49.5, 64.2)	58.2 (49.5, 67.1)	65.9 (55.5, 74.7)	**<0.001**
**12 o'clock**	42.5 (37.0, 48.7)	45.8 (38.5, 56.2)	47.0 (41.9, 63.3)	**<0.001**
**11 o'clock**	42.2 (38.5, 71.2)	43.8 (39.6, 73.8)	45.7 (40.5, 83.0)	**0.041**
**10 o'clock**	39.8 (36.7, 46.0)	40.8 (37.2, 47.3)	43.8 (39.5, 80.3)	**<0.001**
**9 o'clock**	44.2 (40.0, 50.5)	42.3 (38.3, 53.9)	47.3 (39.8, 72.4)	**0.020**
**ASA**				
**15 o'clock**	58.9 (53.9, 62.8)	60.9 (54.7, 67.0)	64.3 (59.7, 70.6)	**<0.001**
**14 o'clock**	79.5 (72.8, 88.4)	85.8 (72.0, 94.5)	90.6 (81.1, 100.6)	**<0.001**
**13 o'clock**	118.7 (113.2, 122.6)	120.8 (115.5, 126.1)	124.1 (118.8, 128.1)	**<0.001**
**12 o'clock**	128.2 (123.3, 131.6)	130.4 (125.1, 134.4)	131.9 (127.5, 136.7)	**<0.001**
**11 o'clock**	126.8 (120.5, 131.4)	128.6 (122.0, 134.0)	128.9 (124.8, 135.3)	**0.009**
**10 o'clock**	117.9 (111.2, 124.4)	118.6 (112.6, 125.4)	119.6 (113.8, 126.4)	0.371
**9 o'clock**	102.1 (95.7, 108.7)	103.2 (97.8, 109.1)	100.9 (96.8, 108.7)	0.450
**Anteversion**				
**femoral head centre**	21.4 (18.8, 24.9)	21.3 (18.0, 24.9)	18.8 (15.8, 21.8)	**<0.001**
**acetabular roof**	12.9 (7.6, 18.6)	12.0 (6.8, 18.8)	11.4 (4.9, 16.0)	0.233

To minimise the potential influence of degenerative changes on morphometric measurements, structure-specific exclusion criteria were applied, i.e. hips with osteophytes were excluded. Sensitivity analysis was performed on hips without acetabular or femoral neck osteophytes to evaluate whether the observed sex differences and morphological patterns persisted.

All tests were two-sided, and p-values <0.05 were considered statistically significant.

## 3 Results

### 3.1 Prevalence of femoroacetabular angles

#### 3.1.1 Alpha-angles

**Fig. 1:**
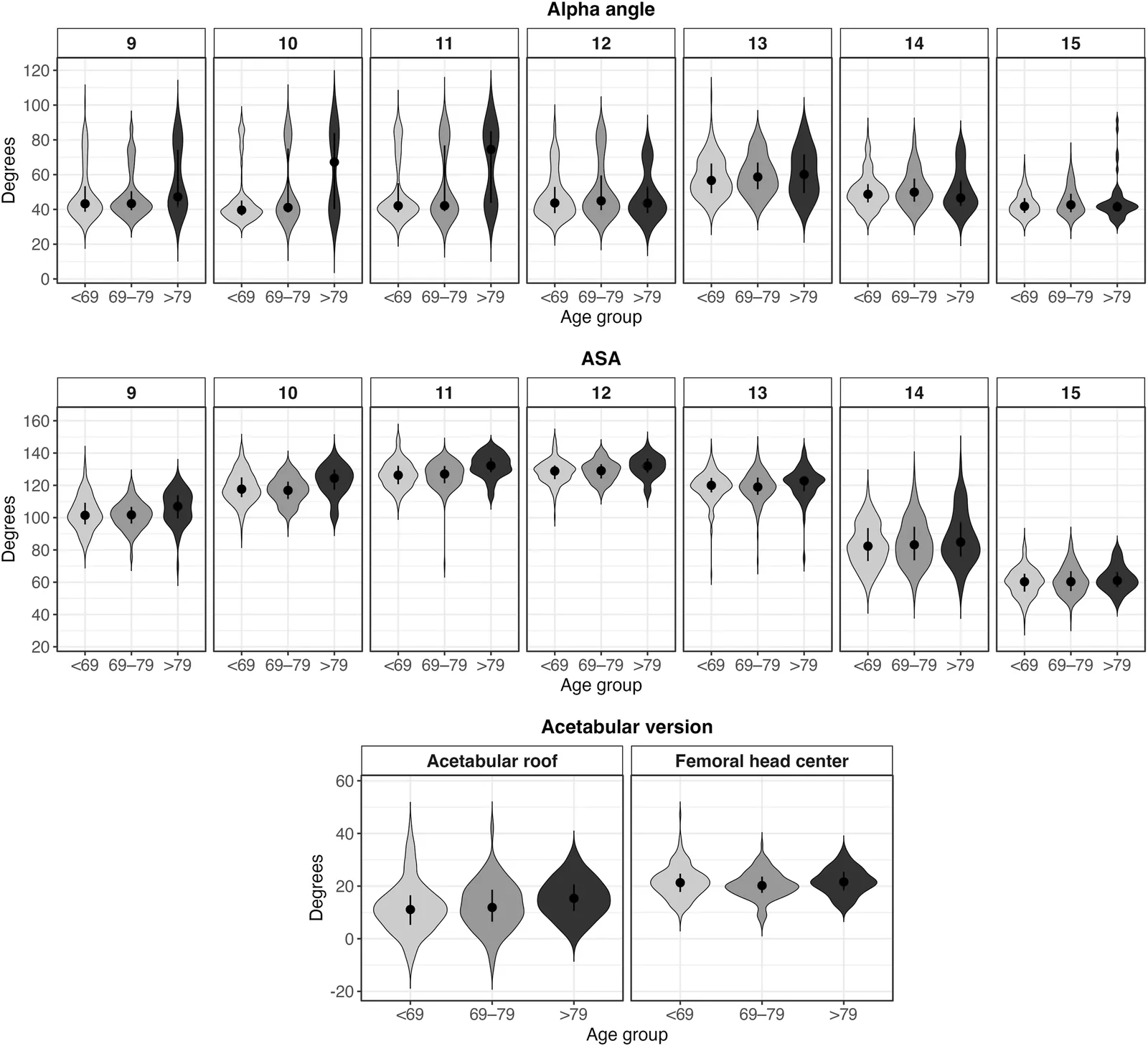
Violin plots showing alpha angles, ASA and acetabular version subdivided into age groups.

Most alpha angles were within the physiological range (<50°), with the only exception at the 13 o'clock position, where the median angle measured 57.6°. Median alpha angles were consistently higher in males than in females across several positions: at 11 o'clock males demonstrated 44.0° compared with 41.3° in females (p = 0.027), at 12 o'clock 46.2° versus 40.8° (p < 0.001), at 13 o'clock 59.7° versus 52.7° (p < 0.001), at 14 o'clock 50.8° versus 46.6° (p < 0.001), and at 15 o'clock 42.8° versus 40.4° (p < 0.001) (Table 2, Figure A.2). Differentiation by age decade revealed the highest median alpha angles in individuals older than 80 years, particularly at 9–11 o'clock (47.2° at 9 o'clock, 67.2° at 10 o'clock, 74.5° at 11 o'clock) (Figure. 1, Table A3). An increase in alpha angles at most clock positions was observed with rising KL grade. At 13 o'clock, values increased from 55.4° in KL 0/1 to 58.2° in KL 2 and 65.9° in KL 3/4 (p < 0.001) (Table 3). In the sensitivity analysis, the gender-specific differences persisted; however, in KL grades, no significant differences were observed (Tables A.4 and A.5).

#### 3.1.2 Acetabular sector angles

ASA were smaller in male participants at multiple positions. At 9 o'clock, males had 99.9° compared with 107.3° in females (p < 0.001); at 10 o'clock 116.5° compared with 123.1° (p < 0.001); at 11 o'clock 126.3° compared with 130.7° (p < 0.001); and at 12 o'clock 128.9° compared with 130.4° (p = 0.002) (Table 2, Figure A.2). After exclusion of hips with acetabular osteophytes, significant sex differences in acetabular sector angles persisted at multiple levels. Women demonstrated significantly higher ASA values at the more caudal levels (9-11 o'clock), whereas men exhibited higher values at cranial levels, most notably at 15 o'clock. Older individuals (>79 years) exhibited higher ASA at several positions, including 12 o'clock, where it reached 131.9° compared with 128.9° in participants younger than 69 years (p = 0.003) (Figure. 1, Table A.3). ASA increased proportionally with rising KL grade, mirroring the pattern observed for alpha angles. At 14 o'clock, ASA rose from 79.5° in KL 0/1 to 85.8° in KL 2 and 90.6° in KL 3/4 (p < 0.001), and at 15 o'clock from 58.9° in KL 0/1 to 64.3° in KL 3/4 (p < 0.001) (Table 3). ASA revealed gender-specific differences in the sensitivity analysis at the 9-12 o'clock position, and at the 12-15 o'clock position for higher KL grades (A.4, A.5).

#### 3.1.3 Acetabular anteversion

Overall median acetabular anteversion measured 21.0° at the femoral head centre and 12.0° at the acetabular roof. Both values differed significantly between men and women (Table 2, Figure A.2). At the femoral head centre, anteversion was 19.9° in males versus 24.2° in females (p < 0.001), while at the acetabular roof it was 10.2° in males and 16.0° in females (p < 0.001). Individuals older than 79 years showed higher anteversion at the acetabular roof, measuring 15.4° compared with 11.1° in participants younger than 69 years (p = 0.006) (Table A.3). Participants with higher KL grades demonstrated significantly lower anteversion at the femoral head centre, decreasing from 21.4° in KL 0/1 to 21.3° in KL 2 and 18.8° in KL 3/4 (p < 0.001) (Table 3).

### 3.2 Correlation of femoroacetabular hip deformities with osteoarthritis

**Fig. 2:**
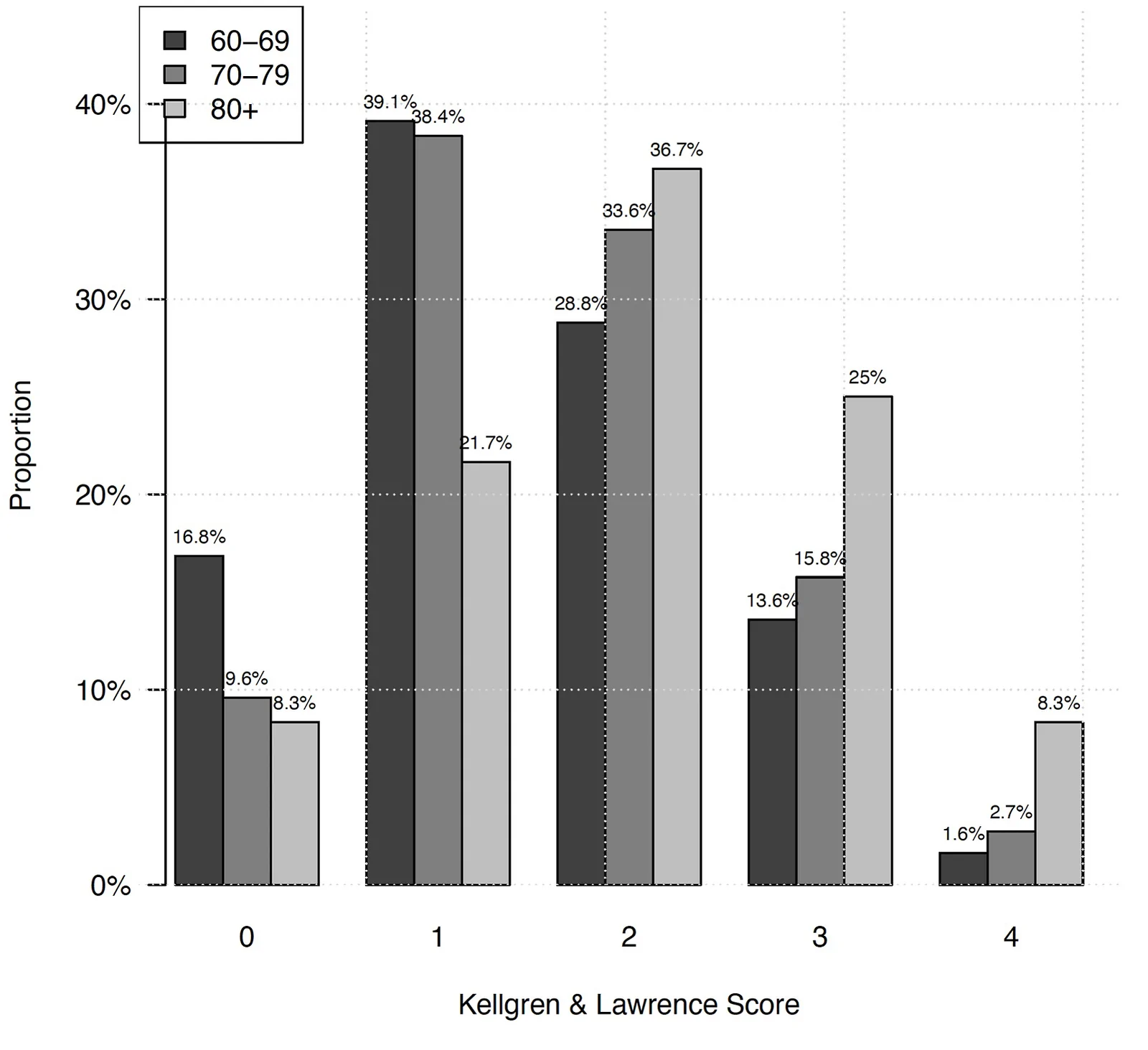
Distribution of KL grades by age groups.

**Fig. 3:**
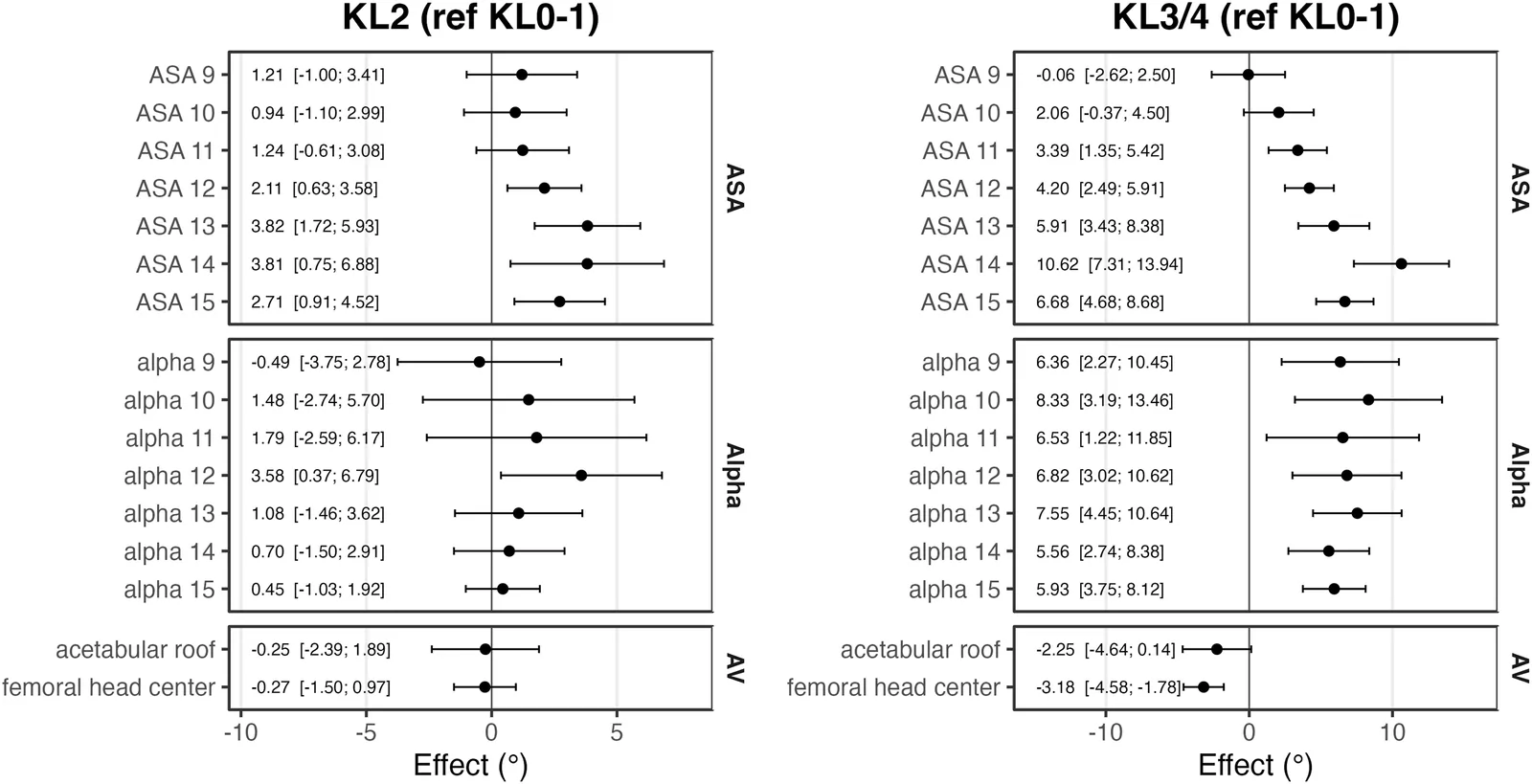
Mixed model for alpha angles, ASA, and anteversion (AV) showing the median deviation (with confidence intervals) of KL 2 and KL 3/4 from the reference (KL 0/1).

Among all hips included, 49% (n = 191) showed no or only doubtful signs of osteoarthritis (KL 0/1), 31.8% (n = 124) had KL 2, and 19.2% (n = 75) were classified as KL 3 or 4, with a continuous increase in higher KL grades among older individuals (Figure. 2, Table A.6). On average, all individuals had very good OHS above 4.5, even in KL 3/4 (Table A.7). According to the median regression model, ASA were significantly higher in individuals with KL 2 and KL 3/4, particularly in the anterior and superior acetabulum, with the largest differences observed in KL 3/4. For instance, at 14 o'clock ASA increased from 79.5° in KL 0/1 to 90.6° in KL 3/4 (p < 0.001) (Figure. 3). Alpha angles were significantly higher only in individuals with severe OA, predominantly at the anterior and superior femoral head–neck junction. At 13 o'clock, values increased from 55.4° in KL 0/1 to 65.9° in KL 3/4 (p < 0.001). Acetabular anteversion at the femoral head centre was significantly smaller in individuals with severe OA, measuring 18.8° in KL 3/4 compared to 21.4° in KL 0/1 (p < 0.001).

## 4 Discussion

In this cross-sectional CT-based study of non-orthopaedic patients aged 60 years or older with asymptomatic hips, we evaluated the prevalence of femoroacetabular impingement–related morphologies and their association with radiographic osteoarthritis severity. Using standardised three-dimensional CT reformations and clock-face–based measurements of alpha angles, acetabular sector angles, and acetabular anteversion, we found that increasing osteoarthritis severity was associated with higher alpha angles and acetabular sector angles, particularly in the anterosuperior region of the hip, as well as with reduced acetabular anteversion at the femoral head centre. These findings support the concept that cam- and pincer-type morphologies represent relevant structural characteristics that are more pronounced in advanced osteoarthritis. Importantly, all individuals included were clinically asymptomatic, indicating that substantial femoroacetabular deformities and radiographic osteoarthritis can exist without relevant hip-related symptoms in older adults. This observation underscores the importance of carefully contextualising CT-detected secondary findings in the clinical setting. Although impingement-associated deformities can act as risk factors for OA, it remains unclear why some patients with impingement deformities develop OA, while others remain asymptomatic until old age without signs of OA. Prior examinations evaluating this issue most often used plain radiographs, sometimes only in the anterior-posterior projection, neglecting the 3-dimensional morphology of the hip. Only a few studies, mostly in Asian and/or younger patients, describe reference values obtained from standardised 3-dimensional reformations. Hence, we compared our 3-dimensional measurements in elderly people with established reference values from other studies, and we further explored correlations between deformities and OA.

The overall hip measurements in our elderly asymptomatic cohort are comparable to those of studies in younger asymptomatic volunteers with a prevalence of FAI deformities; however, an age- and gender-dependent relationship exists.

Fischer et al. examined over 3200 healthy volunteers in a large-scale MRI-based cohort study and found a mean alpha angle of 55° at 12 o'clock. In contrast to our examination (44.3°), this pathological value in asymptomatic volunteers might be explained by better depicting the femoral head contour in reformatted 1 mm slice CT scans instead of non-reformatted 3 mm coronal MRI scans.^23^ Further, the more clinically relevant aspect of the anterosuperior head-neck junction was not evaluated, and the examined individuals had a lower mean age than the volunteers in our study (53 years). Still identical to our study, they observed higher alpha angles in men than women (e.g., 60° vs 53° at the 13 o'clock position). A smaller MRI-based study using radial sequences in 200 asymptomatic young volunteers by Hack et al. analysed the alpha angle in the 15 and 1:30 o'clock position and described higher values in the anterocranial position (40.8° vs 50.2°), similar to our results.^24^ They also found higher alpha angles in men than in women at both anterior and anterosuperior locations (44.0° vs 38.2° and 54.1° vs 47°). Contrary to Hack et al., who examined a group of young, non-symptomatic volunteers (mean age, 29.4 years) using MRI, we detected an age dependence of the alpha angles.^24^

Anda et al. established the ASA at the 15 and 9 o'clock position, examining a small group of patients between 17 and 74 years with CT (ASA 15 o'clock: male 64 ± 6°, female 63 ± 6°, ASA 9 o'clock male 102 ± 8°; female: 105 ± 8°).^25^ Compared with this standard cohort, we detected a modest mean reduction in anterior coverage in our group, but a marked increase in posterior coverage, especially in women. Fuji et al. described the ASA at the 15, 12, and 9 o'clock positions (63.4°; 129.1°; 101.4°) in a control collective of 37 Japanese asymptomatic patients showing a moderately increased coverage anteriorly, more severe at the 12 o'clock position, and reduced in the posterior aspect.^26^

Different control cohorts reported a mean acetabular version at the femoral head centre ranging from 18.2° to 21.5°. In contrast to Yin et al., we detected a significantly increased acetabular version in women.^27^

Higher alpha angles and ASA, as well as lower acetabular version, representing FAI deformities, were correlated with severe OA. The increased alpha angle, combined with a reduced version in male patients, suggests a more common problem with impingement deformities. In the non-orthopaedic cohort, the expression of deformities correlated with the degree of OA. Most prior studies evaluated OA and FAI using radiographs. Thomas et al. prospectively evaluated a female cohort over 20 years and defined an alpha angle above 65° on anterior-posterior radiographs as a predictor for the development of radiographic OA. In this study, the control group had an alpha angle of 46.5°, while the group with radiographic OA had 55.8°. However, when comparing these values, one must consider that females are typically less affected than males.^6^ In a cross-sectional study evaluating senior athletes, Anderson et al. described a common occurrence of FAI with an alpha angle over 50° in 76% of the athletes, but no association with OA.^28^ Furthermore, Wyles et al. described in a retrospective study evaluating OA progression on the contralateral side for cases with THA at a mean age of 47 years, a similar degenerative progression over the following 20 years, comparing FAI and normal hip morphology.^29^ A possible reason could be that patients younger than 65 years with the indication for THA have significantly more often impingement deformities than older patients, where the risk factors of OA are different.

Khanna et al. described an increased alpha angle at the 1:30 o'clock position in a prospective MRI-based study evaluating patients with 2 years of follow-up who had a concern for hip pain. Similar to this study, we found a significant increase in the alpha angle at 13 o'clock in severe OA and in the remaining measured alpha angles, with a median difference of up to 10.5° between KL 0/1 and KL 3/4.

In the setting of secondary findings in radiology, it is crucial to identify and report deformities that may cause symptoms later on,^30^ possibly even before the onset or progression of OA. For this reason, it would be interesting to prospectively investigate the clinical course of asymptomatic hips with femoroacetabular deformities and no or only mild OA in patients of advanced age. The only study investigating this question, a retrospective study, focused on elderly osteoporotic men and relied on radiographs.^5^ They found no correlation between FAI and hip pain in a questionnaire covering the last 30 days.

This study has some limitations. Although standardised pelvic CT measurements were performed, interpolation of osteophytes may induce inaccurate measurements. However, the persistence of sex-specific differences in acetabular sector angles and alpha angles after osteophyte exclusion suggests that these differences reflect inherent morphological variation rather than secondary changes related to degenerative osteophyte formation. A well-established, standardised approach to CT reformations and angle measurements enables reproducible results. Still, reformatting is performed differently in most studies. Some authors correct the pelvic tilt, while others retain the patient-specific position on the CT table, possibly resulting in different acetabular coverage. The skewed distribution of ASA and alpha angles in our study suggests such a source of error. However, the skewed distribution of the same angles in the group with KL 0/1 refutes this assumption. We would rather attribute this distribution of angles to certain femoroacetabular phenotypes than to measurement error, as genetic tests suggest.^31^^,^^32^ However, our observation of higher alpha angles and ASA in OA needs further validation. Furthermore, we excluded patients younger than 60 years to identify FAI morphologies related to long-term osteoarthritic changes and, therefore, potentially excluded severe deformities leading to early OA. As another limitation, not all of our study results, presented as median values, can be directly compared to the literature, which primarily uses mean values. Additionally, femoral torsion could not be accounted for, as oncological CT scans for staging typically end at the pelvic floor.

## 5 Conclusion

In conclusion, this study provides normal values for a 3-dimensional assessment of the acetabulum and the femoral head-neck junction in a population over 60 years of age with asymptomatic hips. The prevalence of femoroacetabular impingement deformities in CT is higher in participants with advanced OA. Especially the anterocranial portion of the hip, which is typically not assessed in standard radiographic imaging, may have a significant influence on the occurrence of OA.

## Guardian/patient's consent

The manuscript includes a statement that all procedures were performed in compliance with relevant laws and institutional guidelines, and that they were approved by the appropriate institutional committee(s); as here, the local ethics committee (EK 530122015, June 7, 2016). This statement contains the date and reference number of the ethical approval(s) obtained. Informed consent was obtained for clinical tests/questionnaires with human subjects. The privacy rights of human subjects are always observed.

## Ethical statement

The manuscript includes a statement that all procedures were performed in compliance with relevant laws and institutional guidelines, and that they were approved by the appropriate institutional committee(s); as here, the local ethics committee (EK 530122015, June 7, 2016). This statement contains the date and reference number of the ethical approval(s) obtained. Informed consent was obtained for clinical tests/questionnaires with human subjects. The privacy rights of human subjects are always observed.

## CRediT author statement

S.F.U.B. – Data Curation, Formal Analysis, Methodology, Resources, Validation, Visualisation, Writing – Original Draft Preparation, Writing – Review & Editing. F.S. – Data Curation, Formal Analysis, Writing – Review & Editing. K.B.– Conceptualisation, Investigation, Data Curation, Formal Analysis, Writing – Review & Editing. P.B. – Investigation, Validation, Writing – Review & Editing. J.-P.K. – Resources, Writing – Review & Editing. R.-T.H. – Resources, Writing – Review & Editing. K.-P.G. – Resources, Writing – Review & Editing. J.G. – Conceptualisation, Data Curation, Investigation, Methodology, Project Administration, Supervision, Validation, Writing – Original Draft Preparation, Writing – Review & Editing. All authors contributed substantially to the conception, drafting/critical review for important intellectual content, final approval of the version to be published, and agreed to be accountable for all aspects of the work in ensuring that questions related to the accuracy or integrity of any part of the work are appropriately investigated and resolved. S.F.U.B. and J.G. take responsibility for the integrity of the work as a whole, from inception to finished article.

## Funding statement

No financial support was received for the conduct of the research and/or preparation of the article.

## Figures and Tables

**A.1:**
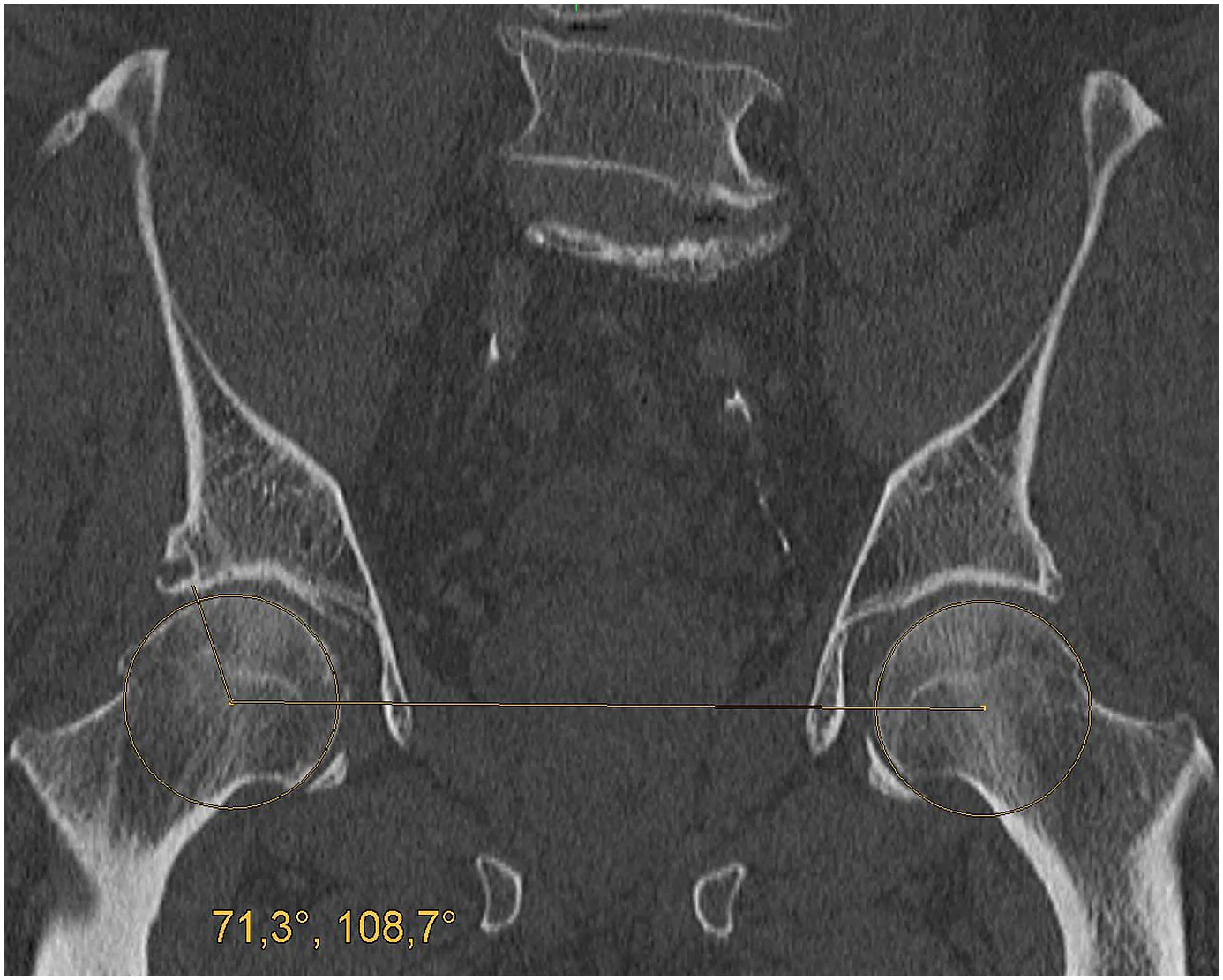
Example of measurement when osteophytes are present. The image below shows a superior ASA measurement at 12 o'clock.

**A.2:**
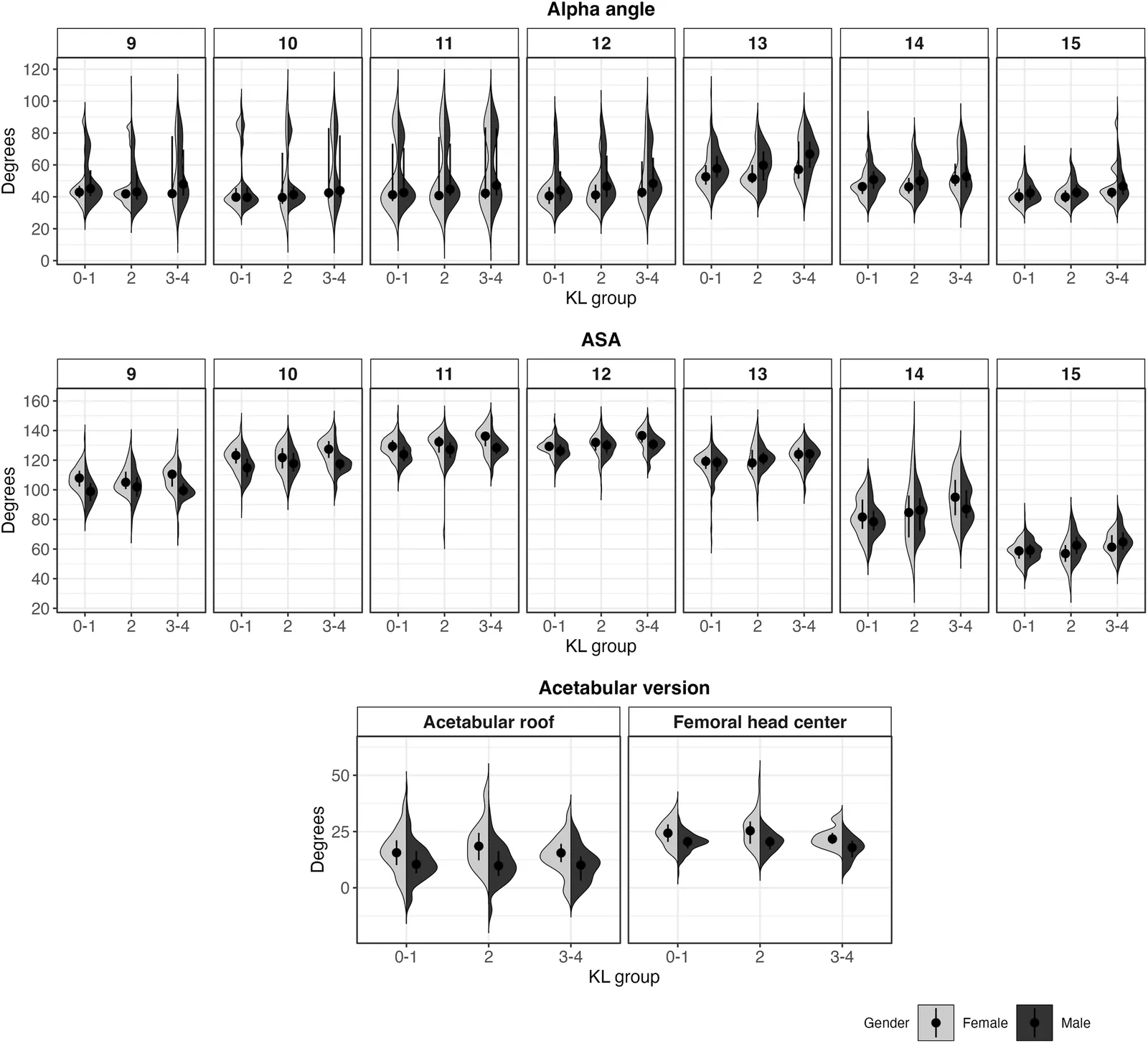
Split violin plots visualising the alpha angles, ASA and anteversion angles for men and women with KL 0/1, 2, and 3/4. The lines within the violins highlight the median, 1st quartile and 3rd quartile.

**A.3:** Distribution of alpha angles, ASA, and anteversion assigned to age groups. Angles are expressed as median with 1st and 3rd quartiles in parentheses.

	<69, N = 184	69-79, N = 146	>79, N = 60	p-value
**Alpha angle**				
**15 o'clock**	41.8 (38.0, 46.5)	42.7 (38.4, 49.0)	41.5 (39.2, 44.5)	0.142
**14 o'clock**	48.7 (44.0, 54.6)	49.9 (44.4, 57.7)	46.6 (41.9, 56.5)	0.326
**13 o'clock**	56.7 (49.4, 66.4)	58.7 (51.6, 67.0)	60.1 (49.3, 71.7)	0.359
**12 o'clock**	43.7 (37.8, 52.9)	44.9 (39.5, 59.6)	43.6 (37.9, 53.1)	0.194
**11 o'clock**	42.1 (38.4, 55.1)	42.2 (39.0, 76.8)	74.5 (43.8, 85.1)	**<0.001**
**10 o'clock**	39.6 (36.5, 45.1)	41.1 (38.2, 75.0)	67.2 (40.2, 83.9)	**<0.001**
**9 o'clock**	43.2 (38.7, 53.3)	43.4 (39.5, 50.5)	47.2 (41.7, 74.3)	**0.003**
**ASA**				
**15 o'clock**	60.3 (54.2, 65.2)	60.3 (54.5, 66.8)	61.0 (56.9, 66.3)	0.291
**14 o'clock**	82.3 (73.2, 93.5)	83.2 (73.6, 94.3)	84.8 (75.9, 97.1)	0.245
**13 o'clock**	120.0 (115.6, 124.6)	119.0 (114.1, 124.9)	122.8 (116.3, 125.6)	0.114
**12 o'clock**	128.9 (123.9, 132.3)	129.1 (124.3, 133.1)	131.9 (127.9, 136.7)	**0.003**
**11 o'clock**	126.3 (120.7, 132.2)	127.0 (121.3, 132.0)	132.2 (128.1, 136.9)	**<0.001**
**10 o'clock**	117.7 (112.7, 124.9)	116.8 (111.6, 122.3)	124.4 (117.3, 129.8)	**<0.001**
**9 o'clock**	101.4 (95.9, 109.2)	101.7 (96.3, 106.7)	107.0 (99.6, 114.0)	**0.004**
**Anteversion**				
**femoral head centre**	21.3 (17.8, 24.8)	20.2 (17.4, 23.6)	21.6 (18.4, 25.5)	0.113
**acetabular roof**	11.1 (5.3, 16.5)	11.9 (6.5, 18.6)	15.4 (10.6, 20.7)	**0.006**

**A.4:** Sex-specific comparison after structure-specific exclusion of osteophytes for sensitivity analysis. Acetabular sector angle (ASA) measurements were analysed after exclusion of hips with acetabular osteophytes, while alpha angle measurements were analysed after exclusion of hips with femoral neck osteophytes. Angles are expressed as median with 1st and 3rd quartiles in parentheses. Group comparisons by sex were performed using the Mann–Whitney *U* test. Sample sizes after exclusion are reported for each variable.

	Total	Women	Men	p-value
Alpha angle (n)	**n = 342**	**n = 120**	**n = 222**	
15 o'clock	41.5 (37.9, 46.2)	40.2 (36.7, 44.4)	42.4 (38.7, 47.1)	**<0.001**
14 o'clock	48.1 (43.4, 53.6)	46.5 (42.2, 50.2)	49.7 (44.0, 54.9)	**<0.001**
13 o'clock	56.0 (49.3, 64.7)	52.5 (47.7, 58.2)	58.7 (50.7, 67.0)	**<0.001**
12 o'clock	43.8 (38.1, 52.8)	40.6 (36.4, 46.3)	46.2 (39.6, 59.4)	**<0.001**
11 o'clock	42.3 (39.0, 68.0)	41.0 (37.5, 67.5)	43.2 (39.7, 68.0)	**0.018**
10 o'clock	40.2 (37.1, 46.0)	39.7 (36.7, 45.2)	40.7 (37.5, 46.0)	0.379
09 o'clock	43.2 (39.0, 49.4)	42.1 (39.0, 45.9)	44.3 (39.4, 54.3)	**0.044**
ASA (n)	**n = 334**	**n = 115**	**n = 219**	
15 o'clock	60.4 (54.8, 65.6)	58.8 (52.6, 62.3)	61.1 (56.2, 67.2)	**<0.001**
14 o'clock	82.8 (73.6, 93.5)	82.4 (72.2, 94.0)	83.0 (74.0, 91.7)	0.581
13 o'clock	120.1 (115.3, 124.7)	119.1 (114.5, 124.0)	120.7 (116.0, 125.1)	0.134
12 o'clock	129.7 (125.0, 133.6)	130.3 (127.0, 134.1)	129.2 (124.0, 133.1)	**0.027**
11 o'clock	127.5 (121.7, 132.9)	130.3 (125.2, 135.2)	126.5 (120.7, 131.1)	**<0.001**
10 o'clock	118.3 (112.9, 124.9)	122.8 (116.4, 128.2)	116.4 (111.0, 122.1)	**<0.001**
9 o'clock	102.2 (96.8, 108.6)	107.0 (101.3, 112.9)	100.4 (95.0, 106.5)	**<0.001**

**A.5:** Hip morphology by Kellgren–Lawrence grade with structure-specific exclusion of osteophytes for sensitivity analysis. Acetabular sector angle (ASA) measurements were analysed after exclusion of hips with acetabular osteophytes, while alpha angle measurements were analysed after exclusion of hips with femoral neck osteophytes. Angles are expressed as median with 1st and 3rd quartiles in parentheses. Comparisons across Kellgren–Lawrence (KL) grades were performed using the Kruskal–Wallis test. Sample sizes after exclusion are reported for each variable.

	KL 0–1	KL 2	KL 3–4	p-value
Alpha angle (n)	**n = 180**	**n = 114**	**n = 48**	
15 o'clock	40.8 (37.4, 46.2)	41.2 (37.9, 45.2)	43.2 (40.1, 48.8)	**0.028**
14 o'clock	48.2 (42.8, 53.3)	47.9 (42.2, 54.0)	47.5 (44.9, 53.4)	0.646
13 o'clock	54.7 (49.2, 63.4)	57.2 (48.7, 64.7)	59.8 (52.2, 69.0)	0.106
12 o'clock	42.8 (37.0, 48.7)	45.8 (38.5, 54.6)	46.3 (40.2, 56.4)	**0.007**
11 o'clock	42.0 (38.5, 66.1)	43.5 (39.5, 71.5)	42.2 (39.3, 54.5)	0.414
10 o'clock	39.7 (36.5, 45.7)	40.5 (37.1, 46.2)	42.0 (38.9, 46.7)	0.069
09 o'clock	43.8 (39.9, 48.4)	42.2 (38.1, 52.1)	43.0 (39.6, 52.5)	0.336
ASA (n)	**n = 168**	**n = 112**	**n = 54**	
15 o'clock	59.1 (54.2, 62.9)	60.4 (54.4, 65.7)	65.1 (60.5, 70.1)	**<0.001**
14 o'clock	80.7 (73.6, 89.0)	84.1 (71.5, 92.1)	87.8 (81.0, 99.6)	**<0.001**
13 o'clock	119.0 (114.2, 122.8)	120.3 (115.4, 125.6)	124.0 (119.8, 126.8)	**<0.001**
12 o'clock	128.4 (124.4, 131.6)	130.4 (124.6, 134.2)	132.7 (128.1, 136.5)	**<0.001**
11 o'clock	127.0 (120.9, 131.7)	128.2 (121.4, 132.9)	128.8 (124.8, 133.6)	0.105
10 o'clock	118.3 (112.8, 124.5)	117.3 (112.5, 125.2)	119.7 (113.8, 125.0)	0.645
9 o'clock	102.3 (96.6, 108.4)	102.7 (97.4, 108.9)	100.8 (96.4, 106.9)	0.535

**A.6:** Radiological features of osteoarthritis according to the Kellgren Lawrence Score. Data are presented as n (%). Percentages refer to the proportion within each grouped Kellgren–Lawrence (KL) category.

Osteoarthritic feature	KL 0–1 (n = 191)	KL 2 (n = 124)	KL 3–4 (n = 75)
Acetabular osteophytes	23 (12.0%)	12 (9.7%)	21 (28.0%)
Foveal osteophytes	153 (80.1%)	87 (70.2%)	59 (78.7%)
Femoral neck osteophytes	11 (5.8%)	10 (8.1%)	27 (36.0%)
Acetabular cysts	4 (2.1%)	0 (0.0%)	5 (6.7%)
Acetabular rim cysts	38 (19.9%)	38 (30.6%)	40 (53.3%)
Impingement cysts	48 (25.1%)	27 (21.8%)	33 (44.0%)
Labral calcification	1 (0.5%)	1 (0.8%)	1 (1.3%)

**A.7:** Oxford Hip Score (OHS) according to Kellgren–Lawrence grade. Values are presented as mean ± standard deviation (SD) with range (minimum–maximum), stratified by Kellgren–Lawrence (KL) grade.

	n	Mean OHS	SD	Range (min–max)
KL 0–1	191	4.87	0.32	2.33 – 5.00
KL 2	124	4.75	0.45	2.33 – 5.00
KL 3–4	75	4.69	0.56	3.00 – 5.00
